# Miniaturized integrated electrochemical sensor using Ag@MoS_2_/graphene oxide aerogel for implantable non-enzymatic glucose monitoring

**DOI:** 10.1038/s41598-025-25570-8

**Published:** 2025-12-01

**Authors:** Haniyeh Shahba, Fatemeh Davar, Navid Nejatbakhsh

**Affiliations:** Department of Chemistry, Isfahan University of Technology, Isfahan, 84156-83111 Iran

**Keywords:** Sensor, Non-enzymatic, Aerogel, Ag nanoparticles, MoS_2_, Reference electrode, Glucose, Biochemistry, Inorganic chemistry

## Abstract

This study reports the successful synthesis of silver-decorated molybdenum disulfide/graphene oxide aerogels (Ag@MoS_2_/GOA) via a combined hydrothermal and freeze-drying approach. This 3D aerogel-based nanocomposite offers a high surface area and porous framework, thereby significantly enhancing the diffusion of glucose molecules and their electrochemical interactions. Electrochemical non-enzymatic glucose sensors were subsequently fabricated by precisely depositing the nanocomposite onto a gold-coated tungsten (W-Au) microelectrode within a highly confined area. To further stabilize the active layer, electropolymerization of pyrrole was employed during the sensor’s construction. This design not only enabled sensor miniaturization but also significantly increased its potential for implantable applications. Furthermore, an integrated reference electrode (IRE) based on Ag/AgCl was developed and seamlessly incorporated into the platform, thereby enhancing to the sensing system’s compactness and self-sufficiency. Surface morphology analyses confirmed the successful and uniform formation of the nanocomposite layer on the electrode surface. Cyclic voltammetry (CV) experiments were conducted in both alkaline (1.0 M NaOH) and neutral (PBS) media, consistently revealing a significant enhancement in glucose oxidation under alkaline conditions. Amperometric measurements across a glucose concentration range of 1–18 mM demonstrated a remarkable sensitivity of 24.70 µA mM^−1^ cm^−2^, a low detection limit of 0.52 µM, a wide linear range, and excellent operational stability. The sensor further exhibited remarkable selectivity, excellent reproducibility, long-term durability, and a relatively fast response time (6–8 s). Collectively, these findings indicate that the Ag@MoS_2_/GOA-based platform, effectively supported by an integrated reference electrode, represents a promising strategy for reliable and enzyme-free glucose detection in complex biological environments.

## Introduction

The majority of bodily tissues rely on glucose (C_6_H_12_O_6_), a key monosaccharide, as their primary energy source. The efficient distribution of glucose in the human body depends on the requirements of specific organs^1^. Although sugars are the primary source of energy for humans, excessive consumption can lead to various ailments, which represent significant global health concerns^2^. According to research, the normal range for blood glucose levels in non-diabetic individuals is between 4.90 and 6.90 mM. A person is diagnosed with diabetes when their blood glucose concentration rises above normal levels^1^. The increasing global prevalence of diabetes, which poses serious health risks and financial burdens, has spurred a significant demand for accurate and efficient glucose sensing technologies in recent years^3^.

For diabetes monitoring, particularly in critical contexts such as dietary intake and fermentation processes, glucose detection is crucial. Despite numerous advancements in this field, glucose detection technology continues to evolve. The pioneering research by Clark and Lyon in 1962 led to the invention of the first glucose sensor. These electrochemical sensors are known as the first generation of blood glucose sensors. Electrochemical blood glucose detection sensors are primarily divided into two categories: enzymatic and non-enzymatic glucose detection sensors^4^. Additionally, they are categorized into generations: first (the most common), second, third, and fourth-generation electrochemical glucose sensors. In the electrochemical glucose detection process, the catalyst plays a critical role. In the first to third generations, the enzyme glucose oxidase serves this function, thus classifying them as enzymatic electrocatalytic sensors. However, enzymatic sensors face inherent limitations, including poor stability under harsh conditions (e.g., extreme pH or temperature), high production costs, stringent storage requirements, and susceptibility to interference from oxygen levels and humidity. These challenges have motivated the exploration of alternative catalysts. In contrast, non-enzymatic sensors, such as fourth-generation systems, leverage advanced catalysts like precious metal nanoparticles, metal sulfides/oxides, novel carbon-based materials, and inorganic–organic composites. These materials offer superior stability, cost-effectiveness, longer shelf life, and broader operational flexibility (e.g., tolerance to extreme environments)^1^. Recent advances in electrochemical sensing have focused on developing a new generation of glucose sensors that completely eliminate the use of glucose oxidases and instead utilize various advanced catalysts for the direct electrochemical oxidation of glucose as biomimetic materials, including precious metal nanoparticles, metal sulfides/oxides, novel carbon-based materials, and inorganic–organic composites^1,3,5–8^. To optimize the performance of these catalytic materials, their deposition on electrode surfaces requires precise control over morphology and interfacial adhesion. Electrochemical polymerization offers distinct advantages for this purpose, enabling the formation of uniform, stable composite layers with strong substrate adhesion-a critical factor for long-term sensor stability under physiological conditions.

Molybdenum disulfide (MoS_2_) typically has a band gap of 1.8 eV and offers a suitable surface area, low coefficient of friction, and superior physicochemical and catalytic characteristics^9^. Non-enzymatic biosensors utilizing MoS_2_ nanosheets have been created; however, due to their low electrocatalytic activity toward glucose, such sensors perform inadequately. Therefore, efforts must focus on enhancing their biosensing performance^10^. Researchers have aimed to improve catalytic properties by focusing on the functional groups of the MoS_2_ surface and combining it with polymers, carbonaceous materials, metal nanoparticles, as well as metal sulfides and oxides. In this context, a non-enzymatic sensor based on CuS/MoS_2_ bimetallic composites was prepared using a one-step hydrothermal process^9^. MoS_2_ nanosheets may be combined with noble metal nanoparticles such as Pt, Ag, Au, Ni, and Cu because noble metals exhibit greater electrocatalytic activity for glucose oxidation^11,12^. Furthermore, fast heterogeneous electron transfer—which significantly boosts electrical conductivity—may result from the interaction between metal nanoparticles and MoS_2_^10^. Silver nanoparticles have also been widely utilized for non-enzymatic detection of blood glucose due to their large surface area and excellent electrocatalytic activity^13^. For instance, Baghayeri et al. used carbon nanotubes (CNT) and Ag NPs to create a non-enzymatic glucose sensor^14^. They demonstrated that the combination of Ag nanoparticles (Ag NPs) and CNT increased the sensor’s long-term stability, sensitivity, and electrode conductivity. Another study investigated non-enzymatic glucose detection using AgNPs/ MoS_2_, fabricated via a hydrothermal process^10^.

Graphene, graphene oxide, activated carbon, and carbon nanotubes are prominent examples of carbon-based compounds that have garnered significant interest in recent scientific research^15^. Carbon-based materials are considered excellent candidates for electrode modification due to their high electron transport, large electrochemical range, high specific surface area, and good chemical stability, rendering them highly suitable for glucose sensing applications^16^. The highly porous structure of graphene oxide aerogel (GOA), composed of interconnected three-dimensional graphene oxide sheets (rich in oxygen-containing functional groups), has drawn considerable interest due to its improved electrical conductivity, large specific surface area, and excellent mechanical strength. Its large pore volume facilitates rapid mass transfer of redox species, and its large specific surface area provides numerous active sites for the sensor’s catalytic process^17^. Graphene possesses a sp^2^-conjugated structure, and the presence of oxygen-containing functional groups disrupts this structure, reducing the electrical conductivity of the material, thereby significantly hindering charge transfer^18^. These features make them a potentially broad and suitable platform for effective nanoparticle placement^17^.

In electrochemical sensing systems, the reference electrode is essential for providing a stable and reproducible potential against which the working electrode operates. Traditional reference electrodes, such as saturated calomel electrodes (SCE) or standard Ag/AgCl electrodes, are often bulky and thus impractical for miniaturized or implantable sensor applications^19^. To address this limitation, integrated reference electrodes (IREs) have been developed, offering compactness and compatibility with microscale devices. Ag/AgCl-based IREs are particularly favored due to their stable electrochemical properties and ease of fabrication. These electrodes have been successfully incorporated into various wearable and implantable biosensors, enhancing their practicality and long-term stability. For instance, microfabricated Ag/AgCl reference electrodes have been utilized in biosensing applications, demonstrating improved performance in miniaturized devices. However, the integration of IREs into non-enzymatic glucose sensors, especially within confined geometries, remains limited. Therefore, incorporating a miniaturized Ag/AgCl-based IRE into the current sensor design not only ensures measurement stability but also contributes to the development of fully integrated and implantable electrochemical sensing systems^20,21^.

In this study, a novel ternary nanocomposite consisting of silver nanoparticles, molybdenum disulfide nanosheets, and graphene oxide aerogel was synthesized via a one-step hydrothermal method and employed as the active material in a non-enzymatic glucose sensing electrode. Unlike conventional binary composites (e.g., Ag/graphene or MoS_2_-based hybrids), our ternary system leverages a hierarchical 3D architecture where MoS_2_ nanosheets prevent AgNP aggregation, GOA ensures mechanical stability and conductivity, and AgNPs provide abundant catalytic sites leading to enhanced synergistic effects for glucose oxidation. The unique 3D architecture of the aerogel, combined with the synergistic catalytic effects of MoS_2_ and AgNPs, enabled a high surface area, excellent electron transfer, and strong electrocatalytic activity toward glucose oxidation. Beyond the promising sensing performance, including high sensitivity, good selectivity, and reproducibility, this work also integrates a miniaturized, on-chip Ag/AgCl reference electrode, paving the way for compact and implantable glucose monitoring devices. The synergistic combination of our ternary composite’s material design and the polymerization deposition fabrication strategy achieves a critical improvement over existing systems, simultaneously enhancing stability, sensitivity, and manufacturability attributes typically compromised in conventional approaches. These combined innovations position the present sensor platform as a promising candidate for next-generation, enzyme-free glucose biosensors with potential real-world biomedical applications.

## Experimental Section

### Chemical reagents

Graphene oxide nanoplatelets (GO NPLs, 99 + %, 3.4–7 nm, 6–10 Layers) and Molybdenum disulfide (MoS_2_, 99.9%, 100 nm) nanopowder were purchased from US Research Nanomaterials, Inc. Silver nanopowder (99.5%, 70 nm) and glucose were purchased from TitraChem and Merck, respectively. Sodium hydroxide (NaOH) was obtained from Mojallali Company. Pyrrole (Py) and poly(styrene sulfonate) (PSS) were purchased from Merck. Phosphate-buffered saline (PBS, pH 7.4) was prepared by mixing stock solutions of NaCl, KCl, KH_2_PO_4_, and Na_2_HPO_4_, all of which were purchased from Mojallali Company.

### Preparation of MoS_2_ suspensions

A 8 mg mL^−1^ initial mass concentration of MoS_2_ nanopowder was achieved by dispersing 160 mg in distilled water. The mixture was stirred at 80°C for 2 h, followed by sonication in a water bath for 4 h using an ultrasonic processor (500 W maximum power, 20% amplitude). The sonication cycle consisted of 6-s “on” and 6-s “off” intervals, with the temperature maintained between 30 and 50°C throughout the process. An alternative method utilized a bath sonicator under similar conditions, with temperature controlled between 40°C and 50°C. Both methods yielded suspensions of comparable quality.

### Synthesis of AgNPs@ MoS_2_/GOA

**Fig. 1:**
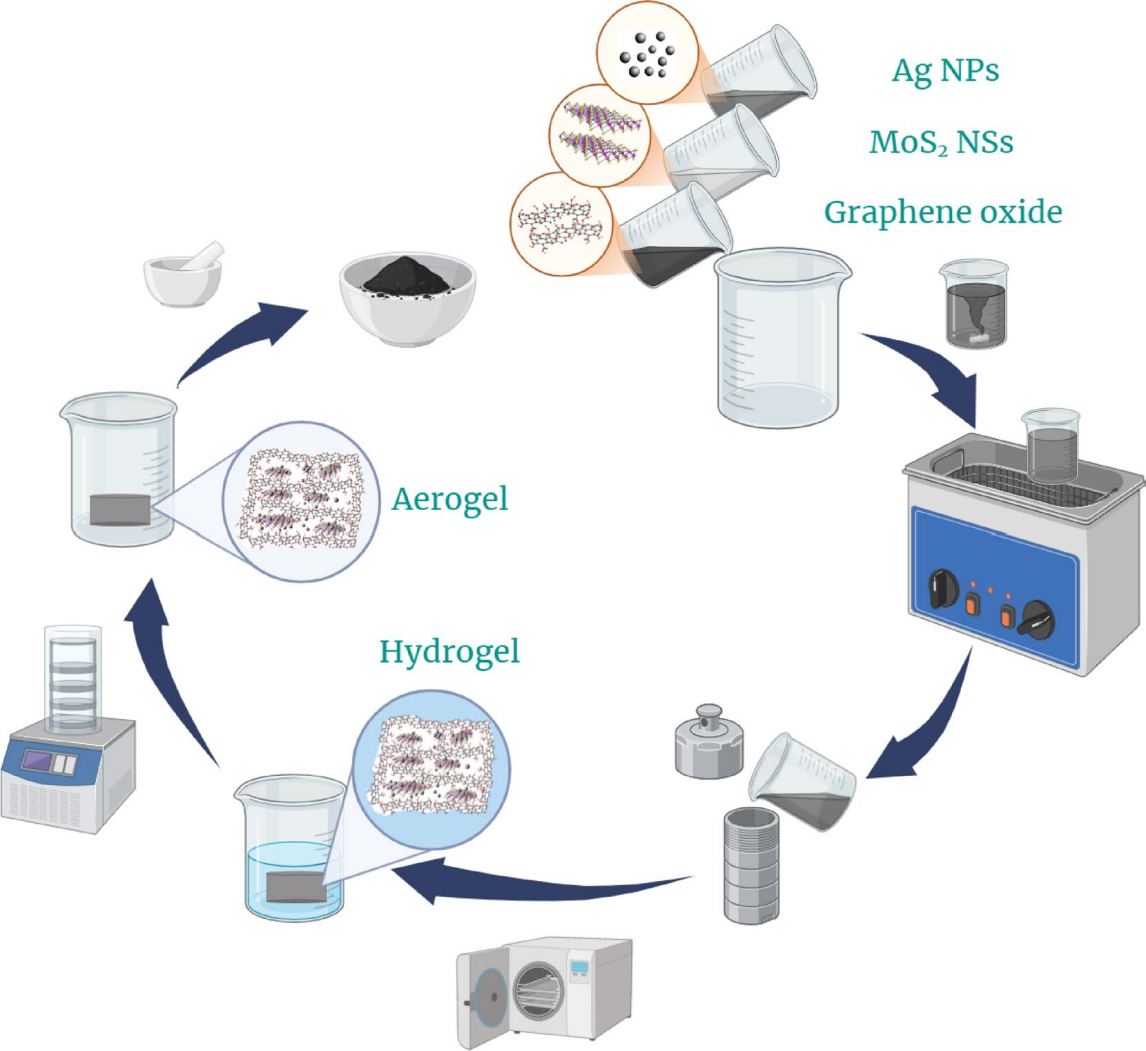
A schematic of the AgNPs@(MoS_2_NSs/GOA) synthesis process.

The synthesis of Ag@MoS_2_/GOA was carried out by mixing 40 mL of MoS_2_ nanosheets (MoS_2_NSs) suspension (2 mg/mL), AgNPs (8 mg/mL), and GO NPLs (3.125 mg/mL). The mixture was sonicated for 1 h. The resulting stable suspension was then transferred to a Teflon-lined autoclave for hydrothermal treatment at 150°C for 5 h. The product was subsequently freeze-dried for 24 h to obtain the final material (See Fig. 1).

### Preparation of the Ag@MoS_2_/GOA-modified electrode

To prepare the Ag@MoS_2_/GOA-modified electrode, 10 mg of the prepared composite was dispersed in 10 mL of PBS (pH 7.4) containing 80.1 mg of poly(styrene sulfonate) (PSS) and 70 µL of pyrrole (Py) monomer. The mixture was sonicated for 1 h to achieve a homogeneous suspension. The electrochemical polymerization and simultaneous deposition of the nanocomposite layer were performed using cyclic voltammetry (CV). The process was carried out in a standard three-electrode system comprising a gold-coated tungsten wire (W-Au, 120 µm diameter) as the working electrode, a platinum wire as the counter electrode, and an Ag/AgCl electrode as the reference. The potential was cycled between -0.3 and + 1.2 V (vs. Ag/AgCl) for one cycles at a scan rate of 50 mV/s. During polymerization, a stable anodic current density of 0.8–1.2 mA/cm^2^ was observed, corresponding to the formation of a conductive poly(pyrrole) (PPy) film that embedded the Ag@MoS_2_/GOA nanocomposite and PSS dopant. This process resulted in the formation of a uniform, adherent, and mechanically stable composite layer with an approximate thickness of 5 µm on the electrode surface.

### Electrochemical measurements of Ag@MoS_2_/GOA/W-AuE

Electrochemical characterization was performed using cyclic voltammetry (CV) with the Ag@MoS_2_/GOA/W-AuE (a gold-coated tungsten wire electrode modified with Ag@MoS_2_/GOA nanocomposites) as the working electrode, a platinum wire as the counter electrode, and an Ag/AgCl electrode as the reference electrode, using an IviumStat potentiostat controlled by IviumSoft software. Amperometric measurements were also conducted using a two-electrode system (working electrode and Ag/AgCl as the reference electrode). Glucose concentration detection was carried out by scanning the potential between − 0.4 V and 0.65 V at a scan rate of 100 mV·s^−1^. Amperometric measurements were performed at 500 mV in NaOH solution. 

## Results and discussion

### Characterization of Ag@MoS_2_/GOA nanocomposite

#### SEM, TEM, EDS and mapping characterization

**Fig. 2:**
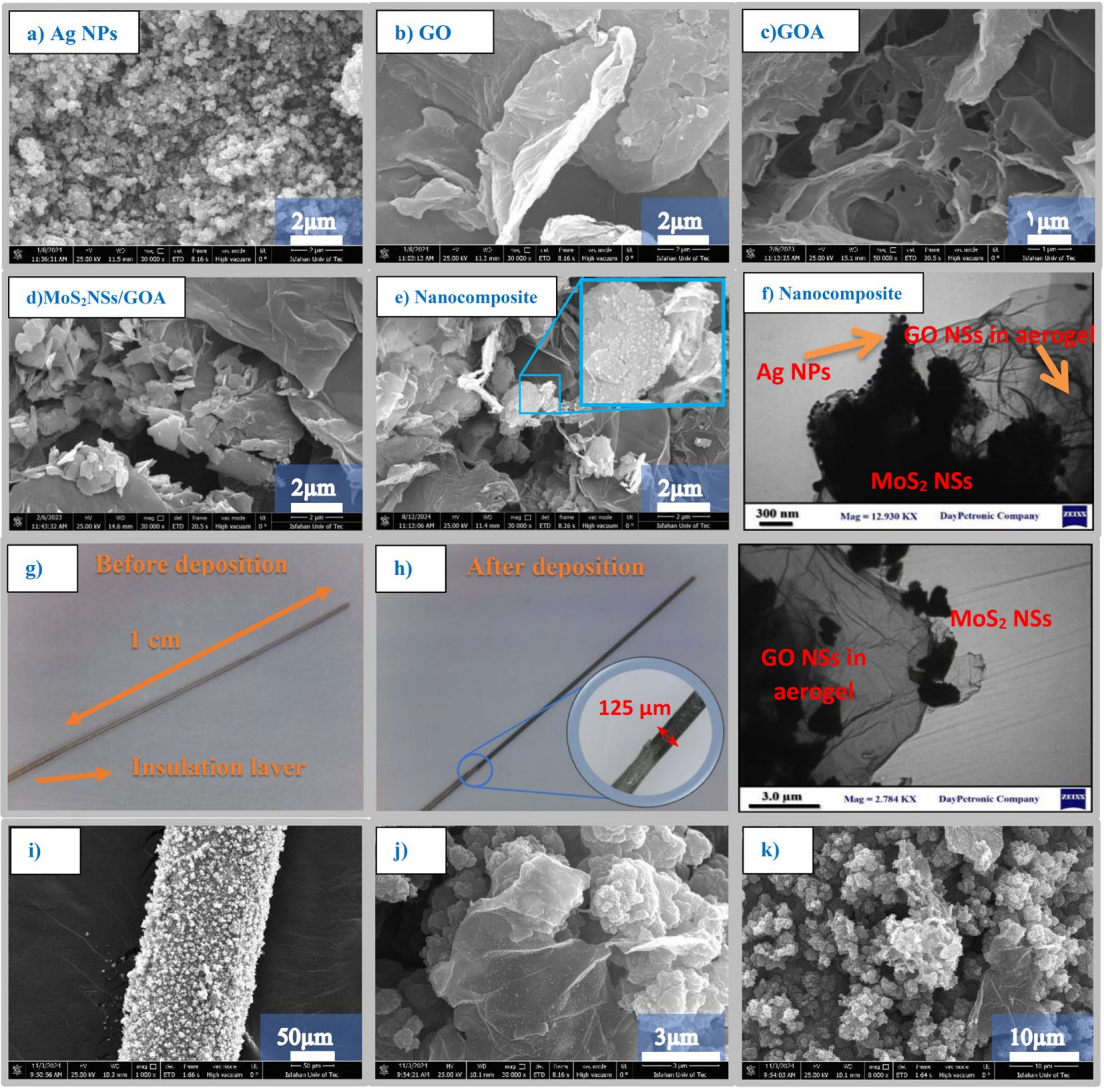
FE-SEM images of (**a**) Ag NPs, (**b**) GO, (**c**) GOA, (**d**) MoS_2_NSs/GOA, and **(e**) AgNPs@(MoS_2_NSs/GOA). (**f**, **g**) TEM image of AgNPs@(MoS_2_NSs/GOA) nanocomposite. (**h**, **i**) real image of Electrode wire before and after deposition nanocomposite and (**j**–**l**) FE-SEM images of AgNPs@(MoS_2_NSs/GOA)/W-Au electrode surface.

Figure 2a–e present the morphological characterization of AgNPs, GO, GOA, MoS_2_/GOA, and Ag@MoS_2_/GOA nanocomposites using field emission scanning electron microscopy (FE-SEM). As shown in Fig. 2b, the graphene oxide sheets form stacked layers, which connect and assemble into a three-dimensional aerogel structure (GOA), depicted in Figs. 1 and 2e. This 3D porous network provides a large surface area and facilitates efficient electron transfer, which is crucial for enhanced electrochemical sensing performance. The Ag@MoS_2_/GOA nanocomposite was synthesized via a one-step hydrothermal method, fostering intimate contact between the components and ensuring uniform dispersion of Ag nanoparticles (AgNPs) on MoS_2_ nanosheets embedded in the GOA matrix. TEM images in Fig. 2f and g clearly display the well-defined architecture of GOA aerogel with MoS_2_ nanosheets and uniformly distributed AgNPs, which serve as active catalytic sites for glucose oxidation.

**Fig. 3:**
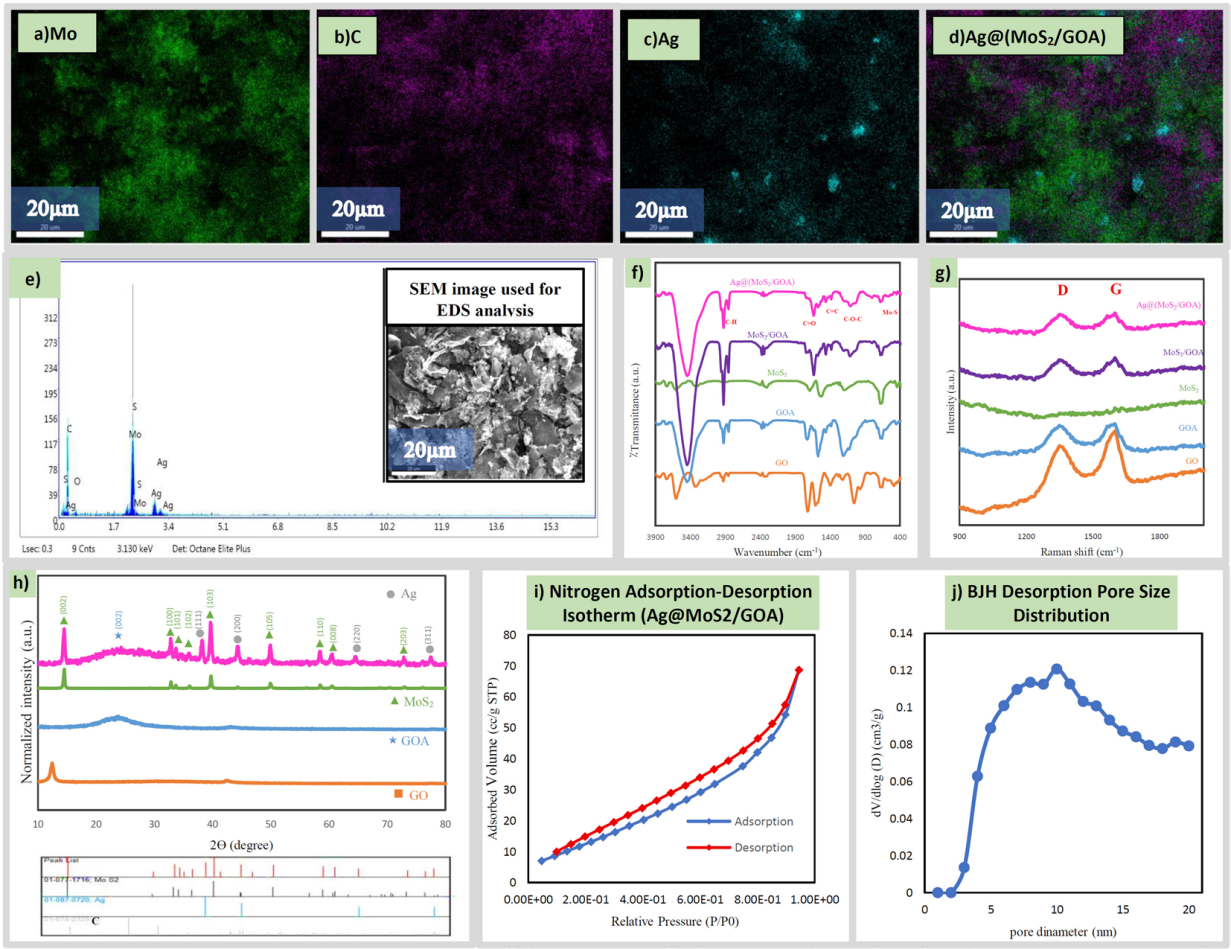
Characterization of the Ag@MoS_2_/GOA nanocomposite: (**a**–**d**) X-ray elemental mapping images showing the distribution of (**a**) Mo, (**b**) C, (**c**) Ag, and (**d**) O. (**e**) EDS spectrum (**f**) FTIR spectra. (**g**) Raman spectra. (**h**) XRD patterns. (**i**) Nitrogen adsorption–desorption isotherm. (**j**) BJH pore size distribution curve.

Figure 2h and i show the electrode wire surface before and after the deposition of the nanocomposite, demonstrating a homogeneous coating. FE-SEM images in Fig. 2j–l, captured after polymerization, further confirm a uniform and porous film formation on the electrode surface, which is advantageous for analyte diffusion and sensor stability. Elemental mapping (Fig. 3a–d) and energy-dispersive X-ray spectroscopy (EDS) analysis (Fig. 3e) confirm the elemental composition and spatial distribution within the nanocomposite. The EDS analysis confirmed the presence of Mo, S, C, O and Ag from MoS_2_, GO aerogel and silver nanaoparticles in the composite. The EDS mapping results (Fig. 3a–d) verify that AgNPs are effectively embedded within MoS_2_ nanosheets, which themselves are well-dispersed throughout the porous cavities of GOA. This hierarchical structure enables synergistic interactions among the components, enhancing electron transport pathways and catalytic activity. The average crystallite size of Ag nanoparticles was estimated to be approximately 70.41 nm using the Scherrer equation based on the XRD diffraction peak, which reflects the crystallite dimension before deposition onto the MoS_2_/GOA surface.

#### FT-IR, Raman. X-ray diffraction and BET analysis

As shown in Fig. 3f, the orange, blue, green, purple, and pink curves correspond to the FT-IR spectra of graphene oxide (GO), graphene oxide aerogel (GOA), molybdenum disulfide (MoS_2_) nanostructures, graphene oxide aerogel composited with MoS_2_, and the Ag@ MoS_2_/GOA nanocomposite, respectively The composite also exhibits a strong and broad O–H stretching vibration band at 3450 cm^−1^, a carboxyl or carbonyl C = O stretching band at 1639 cm^−1^, and an alkoxy O–C–O stretching vibration at 1105 cm^−1^. The prominent peak at 1581 cm^−1^ can be assigned to the carbon double bond^16,22,23^.

Raman spectroscopy was used to study the carbon nanostructure. The Raman spectrum in Fig. 3g clearly demonstrates the formation of graphene oxide aerogel and the presence of MoS_2_ nanostructures in the pink curve, which corresponds to the Ag@MoS_2_NSs/GOA nanocomposite. The orange Raman spectrum represents graphene oxide, which has a D-to-G ratio of 0.84. This ratio increases to 1.004 for GOA in the blue, violet, and pink Raman spectra. In general, the I__D_/I__G_ ratio quantifies the defects in the lattice and the disruption of conjugation. Similar to previous studies^24^, the disorder and conjugation disruption increase with the formation of the aerogel.

Figure 3h displays the XRD patterns of the prepared materials. The orange curve corresponds to graphene oxide (GO), showing a sharp peak at 12.5°, which is attributed to the (001) plane. The blue curve exhibits a broad diffraction peak centered at 2θ = 24°, clearly indicating the formation of graphene oxide aerogel (GOA) with a disordered and porous structure. The green curve shows the characteristic peaks of molybdenum disulfide (MoS_2_) nanosheets at 33° and 58.5°, which correspond to the (100) and (110) planes, respectively. Additional peaks observed at 14.5°, 33.5°, 36°, 39°, 50°, 60.5°, and 73° are indexed to the (002), (101), (102), (103), (105), (008), and (203) planes, in accordance with the hexagonal crystal structure of MoS_2_ (JCPDS#01-073-1508)^25–27^. The curve for the Ag@MoS_2_/GOA composite also shows new peaks at angles of 77.5°, 64.5°, 44.5°, and 38°, in addition to the peaks related to GOA and MoS_2_. These new peaks are attributed to the crystalline structure of silver nanoparticles, confirming the face-centered cubic (fcc) structure of silver nanoparticles (JCPDS#01-087-0720)^28^.

The nitrogen adsorption–desorption isotherm of the Ag@MoS_2_/GOA nanocomposite, presented in Fig. 3i, corresponds to a Type IV isotherm according to IUPAC classification^29,30^, which is a typical behavior of mesoporous materials. The isotherm exhibits a well-defined H3-type hysteresis loop, generally attributed to slit-shaped pores or interlayer voids formed by the aggregation of plate-like particles. This observation is in strong agreement with the FESEM micrographs of the nanocomposite (see Fig. 2), which reveal stacked, layered structures and nanosheet-like morphology, consistent with the formation of interparticle mesopores. The BET surface area, determined using the multi-point method, was 54.78 m^2^/g, which is considered a favorable value for enhancing surface-related electrochemical processes. The corresponding BET constant (C = 17.12) suggests moderate adsorption interactions between nitrogen and the surface. The BJH desorption pore size distribution curve (Fig. 3j) confirms that the majority of the pores fall within the mesoporous range of 5–15 nm. This distribution is responsible for the observed hysteresis loop and reflects a network of interconnected mesopores, where capillary condensation takes place during adsorption and desorption^31^. Due to the non-rigid and open nature of such structures, nitrogen adsorption and desorption do not occur identically, which leads to the characteristic hysteresis behavior. Overall, the porosity analysis supports the presence of a hierarchical porous structure, combining mesopores with smaller microporous regions, which is highly beneficial for improving mass transport, electrolyte penetration, and enhancing the sensitivity of the electrochemical glucose sensor^32^.

### Electrochemical characterization for non-enzymatic glucose detector

#### Cyclovoltammetry analysis

**Fig. 4:**
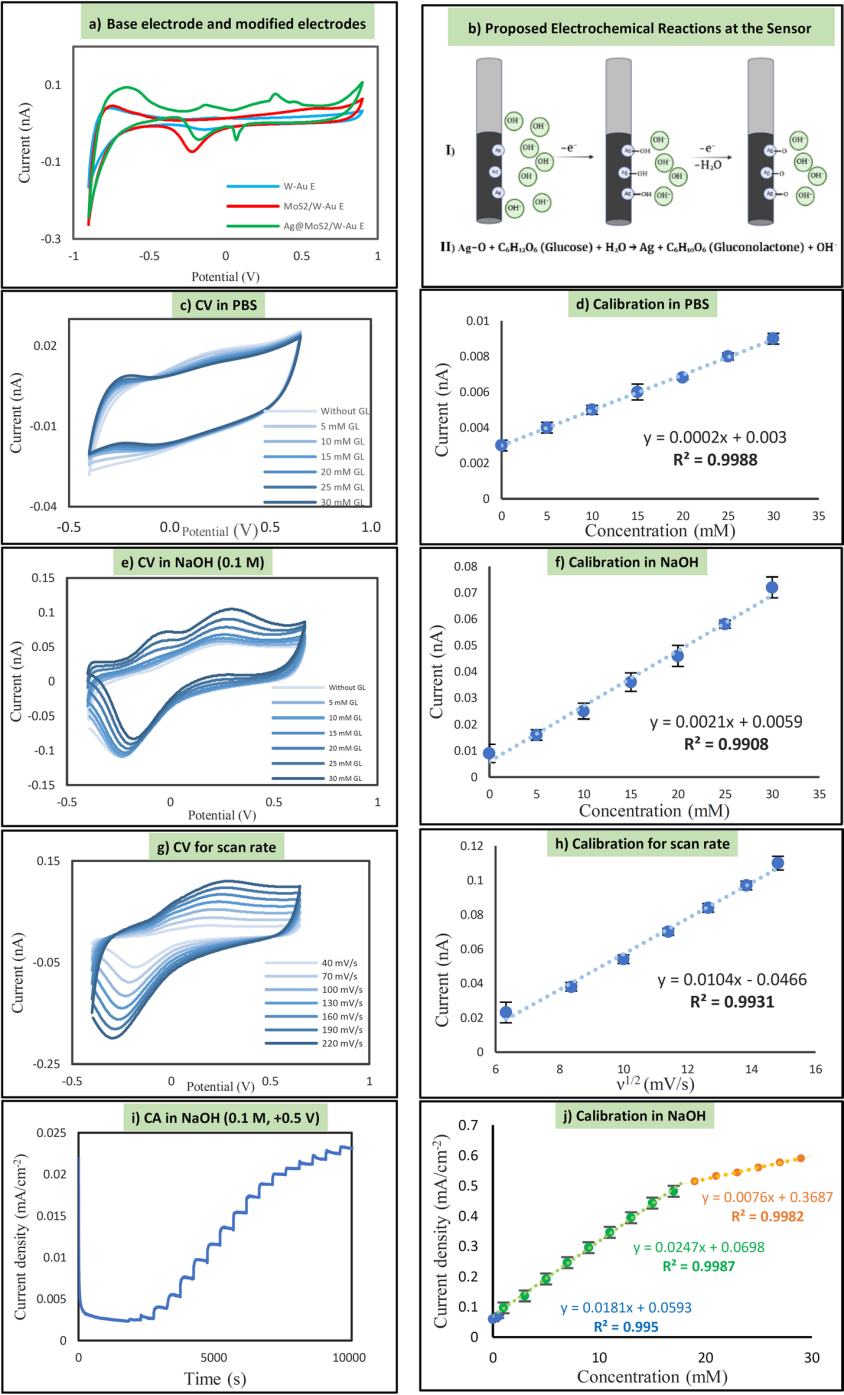
Electrochemical characterization of the glucose sensor. (**a**) CV curves of different modified electrodes in the presence of glucose. (**b**) Schematic of proposed glucose oxidation mechanism. CV responses of the Ag@MoS_2_/GOA sensor in (**c**) PBS and (**e**) 1.0 M NaOH with increasing glucose concentration. Corresponding calibration plots in (**d**) PBS and (**f**) NaOH. (**g**) CVs at different scan rates. (**h**) Linear relationship between peak current and square root of scan rate. (**i**) Amperometric (I-t) response to successive glucose additions. (**j**) Calibration curve derived from (**i**); error bars represent ± SD (n = 3).

Figure 4a presents a comparative analysis of the cyclic voltammetry (CV) curves for the base electrode and modified electrodes in the presence of glucose. The base electrode (W/Au) demonstrates the lowest current response, reflecting minimal electrochemical activity. This suggests a limited number of catalytic sites, making it an appropriate reference for evaluating subsequent modifications. The enhanced current response observed in the MoS_2_-modified electrode can be attributed to its large surface area and electroactive properties. MoS_2_ facilitates charge transport and promotes redox reactions, thereby improving glucose oxidation. Moreover, the defect-rich edges and sulfur vacancies of MoS_2_ serve as additional electroactive sites, facilitating glucose adsorption and electron transfer, thereby complementing the catalytic role of AgNPs.

In the MoS_2_ + Ag-modified electrode, the further increase in current response indicates improved electrical conductivity and enhanced electrocatalytic activity toward glucose oxidation. Silver nanoparticles contribute to faster electron transfer kinetics due to their high electrical conductivity and intrinsic catalytic properties, providing additional active sites for glucose detection. The appearance of new peaks may correspond to silver oxidation/reduction processes involved in glucose electrooxidation. While MoS_2_ enhances catalytic activity through its layered structure and active edges, silver nanoparticles serve as catalytic centers, further improving electron transfer and glucose sensitivity. MoS_2_ not only provides a high surface area support but also mediates electron transfer from glucose molecules to AgNPs, enhancing the overall electrocatalytic efficiency.

The combination of MoS_2_ and Ag exhibits a synergistic effect, maximizing the electrochemical response in the presence of glucose. The observed peak shifts and intensifications indicate enhanced electron transfer and improved glucose oxidation efficiency, confirming that each modification progressively enhances the electrode’s performance for glucose sensing. Additionally, the layered MoS_2_ structure may stabilize intermediate species formed during glucose oxidation, preventing aggregation of AgNPs and promoting sustained electron flow.

Electrochemical analysis of Ag@MoS_2_/GOA/W-AuE was conducted to evaluate its electrocatalytic performance for glucose oxidation using cyclic voltammetry (CV) (Fig. 4c–h) and chronoamperometry (Fig. 4i, j). The results indicate excellent catalytic activity of the Ag@MoS_2_/GOA/W-Au electrode. Comparing the CV results in PBS solution (pH 7.0) with those in a 1.0 M NaOH aqueous electrolyte solution shows that the current increase in NaOH solution is significantly greater than that in PBS solution. Therefore, electrochemical detection of glucose using a non-enzymatic method requires alkaline conditions, which were used for subsequent electrochemical measurements. It is expected that in non-enzymatic sensors, where glucose oxidase enzyme is absent, inorganic catalysts require hydroxide ions to detect glucose. As a result, the biosensor exhibits higher sensitivity in NaOH solution. In PBS solution, the hydroxide ions resulting from water dissociation are less abundant and do not contribute significantly to increasing sensitivity.

The CV analysis of the Ag@MoS_2_/GOA/W-Au electrode was conducted to study the effect of increasing glucose concentration, as shown in Fig. 4c and e for neutral and alkaline environments, respectively. The electrode was placed in a 1.0 M NaOH stock solution at a potential of 0.5 V, and the glucose concentration was gradually increased. The selection of the -0.04 V peak as the quantitative glucose detection signal, which is based on increasing glucose concentrations, was made possible by the positive scan. This scan revealed a significantly higher current at the -0.04 V peak compared to the 0.275 V peak. As the analyte concentration increases, the number of species that lose electrons increases, leading to more electrons circulating in the circuit and a corresponding increase in the measured current^10,33^. Based on the results from Fig. 4d and f shows the calibration curve of the Ag@ MoS_2_/GOA/W-Au electrode for glucose detection. The sensor exhibited a well-defined and concentration-dependent electrochemical response toward glucose in the range of 0.0–30.0 mM. As the glucose concentration increased, the current response of the sensor increased accordingly, confirming its capability for reliable glucose detection. Figure 4g shows the CV analysis results of the AgNPs@MoS_2_/GOA/W-Au electrode at scan rates of 40–220 mV/s. As evident from Fig. 4h, the peak current increases as the electrode scan rate increases.

It is likely that silver nanoparticles are initially oxidized to AgOH and AgO species in the presence of hydroxide ions. This process creates favorable conditions for the oxidation of glucose, during which glucose releases electrons, resulting in current generation (Fig. 4b).

Silver-based electrocatalysts exhibit a distinct mechanism in glucose oxidation compared to transition metals like Ni, Cu, and Co. Unlike these metals, which directly participate in glucose oxidation via the formation of stable metal-glucose complexes, silver acts primarily as an electron mediator rather than a direct catalytic site. The electrochemical oxidation of silver in an alkaline medium lead to the formation of AgO, which serves as an electron acceptor in the oxidation of glucose. This process differs fundamentally from Ni, Cu, and Co, where metal oxyhydroxides (e.g., NiOOH, CuOOH) directly oxidize glucose through redox cycling. The absence of strong metal-glucose interactions in silver-based systems reduces unwanted side reactions, contributing to higher stability and selectivity in non-enzymatic glucose sensing applications. This distinction makes silver a promising candidate for long-term electrochemical sensing, particularly in implantable and continuous monitoring devices^33,34^. Cyclic voltammetry analysis were performed in triplicate at each glucose concentration to ensure repeatability. The calibration curve was constructed using the average current response of the three measurements, and the error bars shown in the figure represent the standard deviation, reflecting the variability between repeated experiments.

(A) In PBS (without OH^−^):
  Glucose oxidation (weaker): C_6_H_12_O_6_ → C_6_H_12_O_7_ + 2H^+^+2e^−^
  Water hydrolysis: 2H_2_O → 2H_2_ + O_2_.
  Silver-assisted electron transfer (same): Ag + e^−^  → Ag^+^

(B) In Alkaline Environment (OH^−^ present):
  Glucose oxidation with OH^−^: C_6_H_12_O_6_ + 2 OH^−^  → C_6_H_12_O_7_ + 2e^−^  + H_2_O.
  Silver-assisted electron transfer: Ag + e^−^  → Ag^+^

**Increased Electron Transfer in Alkaline Medium:** In an alkaline medium, the presence of hydroxide ions (OH^−^) facilitates a more efficient oxidation of glucose. The oxidation reaction of glucose in an alkaline medium involves the direct interaction of glucose with OH^−^ ions, which significantly increases the rate of the reaction. As a result, more electrons are released during the glucose oxidation process, leading to a higher current.

**Role of Hydroxide Ions (OH**^**−**^**):** In alkaline solutions, OH^−^ ions play a critical role in enhancing the rate of glucose oxidation by providing additional reaction sites on the electrode and facilitating the oxidation process. This leads to more electrons being transferred to the electrode, which directly correlates with an increase in the peak current observed in the cyclic voltammetry measurements. Additionally, OH^−^ ions might help prevent the passivation of the electrode surface, which can sometimes occur in neutral or acidic conditions, reducing the efficiency of electron transfer. Therefore, in alkaline conditions, the overall reaction proceeds more efficiently, yielding higher current.

**More Peaks in the CV Plot:** The additional peaks in the cyclic voltammetry plot in an alkaline medium are may due to multiple electrochemical reactions occurring simultaneously at different potential windows, which is typical in complex systems with multiple active species or reaction pathways. These peaks may represent:

- Glucose oxidation and reduction at different electrode potentials, with oxidation occurring at one potential and reduction occurring at another.
- Side reactions, such as the formation and reduction of intermediate species (like glucose oxidation products), which may have their own characteristic redox potentials.
- Hydroxide ion interactions, where OH^−^ may participate in various electrochemical reactions alongside glucose oxidation.
- The multiple peaks indicate that various electrochemical processes are taking place, and these are more likely to occur in an alkaline medium because of the increased number of possible redox reactions between glucose, the electrode surface, and hydroxide ions.

**Higher Sensitivity and Resolution:** In alkaline solutions, the higher concentration of OH⁻ ions and the more efficient electrochemical processes contribute to an overall increase in the current and a more detailed electrochemical response. This promotes the resolution of peaks in the CV plot, allowing for the detection of multiple reaction steps that may not be as distinguishable in PBS or neutral solutions. It is probable that silver nanoparticles are first oxidized to AgOH and AgO species in an environment containing hydroxide ions, and then oxidize glucose, converting it to gluconolactone, which is itself reduced.

The enhanced performance of the Ag@MoS_2_/GOA sensor in an alkaline medium can be attributed to the increased availability of hydroxide ions (OH⁻), which facilitate the oxidation of glucose. In alkaline conditions, the silver nanoparticles are oxidized to AgOH and AgO species, which act as active sites for glucose oxidation. This process not only increases the rate of electron transfer but also prevents the passivation of the electrode surface, which is a common issue in neutral or acidic environments. The presence of OH⁻ ions also stabilizes the intermediate species formed during glucose oxidation, leading to a more efficient and stable electrochemical response.

#### Chronoamperometry analysis of the sensor with coated working electrode

Figure 4a shows the chronoamperometric spectrum of the platinum electrode in a basic alkaline solution (0.1 M NaOH). As is evident, the gradual addition of glucose to the solution causes an increase in the current. he stepwise increase in current density corresponds to the successive addition of glucose every 60 s, and the trend of increasing current density maintains a broad linear relationship in the glucose concentration range of 0.005–30.0 mM. In fact, the detection limit represents the lowest concentration of a solute that an analytical system can reliably discriminate. Additionally, the sensitivity of the sensor is determined by the slope (m) of the calibration equation^35^.

The electrode shows a linear response between 1.0 and 18.0 mM (equivalent to 18 to 324 mg/dL). The calculated sensitivity for this electrode is 24.70 μA mM^−1^ cm^−2^, and with a limit of detection (LOD) of 0.52 μM (The LOD was calculated as 3σ_b_/S, where σ_b_ represents the standard deviation of the blank signal obtained from repeated measurements (n = 10). The error bars in the calibration plot indicate the standard deviation from three repeated measurements at each glucose concentration). Sensitivity is defined as the slope of the curve generated when the measurement results are plotted against the determined concentrations. This definition of sensitivity corresponds to the slope of the analytical calibration curve. The lower limit of detection is defined as the level below which detection is impossible with a certain degree of certainty^36^. Moreover, the response time of the sensor, estimated from the amperometric I–t curve, was approximately 6–8 s. This value was determined as the time required to reach 90% of the steady-state current following each glucose addition. The linear range, detection limit, and sensitivity of comparable materials that have been developed and reported elsewhere are contrasted in Table 1.

**Table 1:** performance comparison between the created non-enzyme glucose biosensor AgNPs@(MoS_2_NSs/GOA)/W-AuE and other non-enzyme glucose biosensors.

Modified Electrodes	Method	Linear range	Sensitivity	Detection Limit	Ref
Ag–PANI/rGO	CV	0.1–50 μM	–	0.79 (µM)	Hu et al.^1^
AuNC(c10)/PPyNW	CV	0.05–10 mM	14.5 (µA mM^−1^ cm^−2^)	48.2 (µM)	Preechakasedkit et al.^2^
AgNPs/MoS_2_	CV	1.0–15 mM	46.5 (µA mM^−1^ cm^−2^)	1.0 (µM)	Bakhshi et al.^3^
rGO	CV	0.2–10 mM	19.17 (µA mM^−1^ cm^−2^)	1.901 (µM)	Govindaraj et al.^4^
Cu (II)/rGO/SPCE	CV Amperometry	0.1–12.5 mM	171.95 (µA mM^−1^ cm^−2^)	65 (µM)	Hung^5^
CuS/MoS_2_	CV Amperometry	0.1–11 mM	252.71 (µA mM^−1^ cm^−2^)	1.52 (µM)	Aygun et al.^6^
Cu/Pani/MoS_2_	CV Amperometry	0.1–11 mM	69.82 (µA mM^−1^ cm^−2^)	1.78 (μM)	Yang et al.^7^
AgNPs@(MoS_2_NSs/GOA)	CV Amperometry	0.005–1mM 1–18 mM 18–30 mM	18.10 (µA mM^−1^ cm^−2^) 24.70 (µA mM^−1^ cm^−2^) 7.60 (µA mM^−1^ cm^−2^)	0.72 (µM) 0.52 (µM) 1.71 (µM)	This work

### Stability and reproducibility of AgNPs@MoS_2_/GOA/W-AuE

**Fig. 5:**
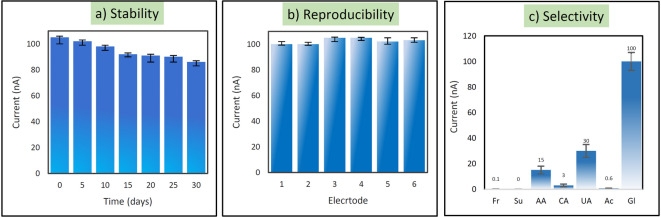
(**a**) Long-term stability of AgNPs@(MoS_2_NSs/GOA)/W-AuE sensor for glucose sensing evaluated over 30 days. Bar heights represent the average current responses obtained from repeated measurements (n=3). (**b**) Reproducibility of AgNPs@(MoS_2_NSs/GOA)/W-AuE sensor in 0.1 M NaOH solution containing 5.0 mM glucose. The bar heights represent the average current responses from multiple repeated measurements, with error bars indicating standard deviation. (**c**) Study of biosensor selectivity for glucose in the presence of interfering species including fructose, sucrose, citric acid, ascorbic acid, acetaminophen, and uric acid. Bar heights represent the average current responses from three (n = 3) repeated measurements, with error bars showing standard deviation.

Another crucial factor in assessing the lifespan and effectiveness of a glucose biosensor is its stability. The stability of the AgNPs@MoS_2_/GOA/W-AuE biosensor was investigated over a period of 30 days, as shown in Fig. 5a, with a stability of 86% observed for the sensor. It exhibited significantly higher stability for glucose detection compared to other non-enzymatic sensors. To evaluate the repeatability of the Ag@MoS_2_/GOA)/W-AuE sensor, the response of six distinct electrodes to a solution with a constant glucose concentration of 5.0 mM was measured. The relative standard deviation (RSD) of 2.20%, as shown in Fig. 5b, indicates that the reproducibility of the Ag@(MoS_2_/GOA)/W-AuE biosensor is excellent.

### Selectivity of Ag@MoS_2_/GOA/W-AuE

To evaluate the selectivity of the designed non-enzymatic glucose sensor, several potential interfering species commonly present in interstitial fluid were tested, including fructose, sucrose, ascorbic acid, citric acid, uric acid, and acetaminophen. The concentrations of these compounds were selected to approximate or exceed their physiological levels in interstitial fluid: 50 µM fructose, 100 µM sucrose, 50 µM ascorbic acid, 200 µM citric acid, 300 µM uric acid, and 10 µM acetaminophen. For comparison, 5 mM glucose was used. Human blood glucose levels typically range from 4.9 to 6.9 mM (88.3 to 124.3 mg/dL), and interstitial glucose levels follow similar trends, though slightly delayed in dynamics^10^. Chronoamperometric measurements were performed at a fixed potential of + 500 mV (vs. Ag/AgCl), a value optimized for the anodic oxidation of glucose on the AgNPs@(MoS_2_NSs/GOA)-modified electrode. Based on the literature, the oxidation potentials of ascorbic acid and uric acid lie between + 200 to + 450 mV vs. Ag/AgCl, while glucose typically oxidizes around + 450 to + 550 mV, depending on the electrode material.

The sensor exhibited negligible current responses to fructose, sucrose, and acetaminophen, indicating excellent selectivity. Citric acid induced only a minor current increase (~ 3 nA), which may be attributed to weak electrochemical activity or complexation effects. Ascorbic acid and uric acid caused moderate current responses of ~ 15 nA and ~ 30 nA, respectively—corresponding to only 15% and 30% of the glucose signal (100 nA). These limited responses are likely due to their oxidation potentials partially overlapping with the applied working potential (Fig. 5c).

Overall, the sensor demonstrated a distinct and dominant current response to glucose, confirming its high selectivity even in the presence of multiple physiologically relevant interferents at biologically realistic concentrations.

### Integration of the Ag/AgCl reference electrode layer

**Fig. 6:**
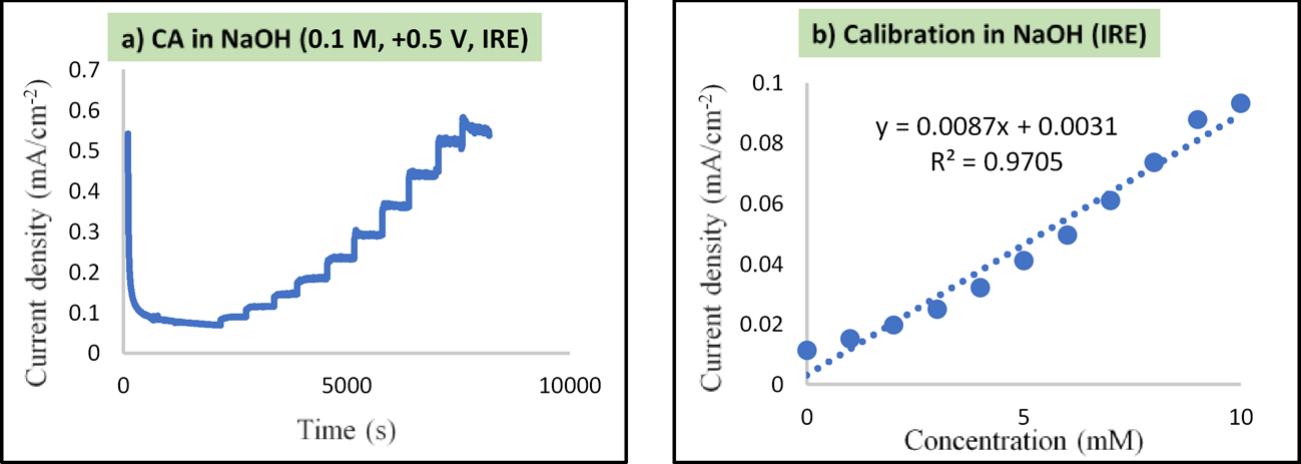
Chronoamperometric analysis of the miniaturized glucose sensor equipped with an integrated Ag/AgCl reference electrode (IRE). (**a**) Current responses to successive glucose additions (0–10 mM) in 0.1 M NaOH at + 500 mV; (**b**) Corresponding calibration plot showing a linear range of 0–10 mM with a sensitivity of 8.70 μA mM^−1^cm^−2^ and R^2^ = 0.97.

To ensure accurate and stable potential control in the electrochemical sensing system, a planar Ag/AgCl integrated reference electrode (IRE) was incorporated directly onto the gold-coated tungsten substrate. This integration eliminates the need for an external reference electrode, enabling full device miniaturization and enhancing the applicability of the sensor in wearable and implantable formats. Chronoamperometric (CA) measurements were conducted using the IRE to assess its performance. As shown in Fig. 6a, successive additions of glucose into 0.1 M NaOH resulted in distinct and rapid current responses, confirming the stability and functionality of the integrated reference system. The corresponding calibration curve (Fig. 6b) demonstrated a linear relationship over the concentration range of 0 to 10 mM, with a sensitivity of 8.70 μA mM^−1^ cm^−2^ and a detection limit of 1.5 μM (based on a signal-to-noise ratio of 3). The regression coefficient (R^2^ = 0.97) confirms the reliability of the sensor’s output when using the IRE. These results validate the successful integration of the Ag/AgCl reference layer and its suitability for stable, accurate, and miniaturized electrochemical glucose sensing, providing a strong basis for further real-sample analysis (Table 2).

**Table 2:** Comparison table of the proposed glucose sensor with other similar sensors.

Sensor composition	Sensitivity (µA·mM^−1^·cm^−2^)	Detection limit (µM)	Linear range (mM)	Performance stability (30 days)	Reproducibility (RSD)	Structural stability	Specific applications	References
AgNPs@(MoS_2_NSs/GOA) nanocoposite	24.7	0.52	0.005–31	86% retention	2.20%	High	Implantable biomedical applications	This work
Colloidal AgNPs on MoS_2_	9044.6	0.03	0.1–1	80% retention	3.10%	Moderate	Point-of-care diagnostics	Sabaghi et al.^8^
NiO-decorated MoS_2_ nanosheets	2310	0.1	0.001–5	78% retention	4.50%	High	Wearable sensors	Sharma et al.^9^
Co_3_O_4_/reduced graphene oxide (rGO) nanohybrid	839.3	0.5	0.001–2	75% retention	5.00%	Moderate	Non-invasive glucose monitoring	Van^10^
Carbon nanodots (C-dots)/CuO nanocomposites	5300	0.5	0.001–1.1	82% retention	3.80%	High	Continuous glucose monitoring (CGM)	Liu et al.^11^

### Real sample analysis

To evaluate the practical applicability of the fabricated electrochemical glucose sensor, real serum samples were analyzed, and the results were compared with those obtained from a commercial handheld glucometer. The detection of glucose in actual biological fluids is crucial for validating the sensor’s performance under complex physiological conditions, where various interfering species may coexist. As summarized in Table 3, four human serum samples were tested. In each case, glucose concentration was measured using both methods. The results obtained with the developed sensor showed close agreement with those from the commercial device, confirming its reliability and potential for real-sample monitoring. All measurements were performed in triplicate (n = 3), and the sensor exhibited excellent repeatability with RSD values ranging from 0.81 to 1.30%. These findings underscore the robustness and analytical precision of the sensor in real-world applications.

**Table 3:** Comparison of glucose concentrations in real serum samples using the proposed sensor and a commercial handheld glucometer (n = 3).

Sample	Handheld glucometer (mmol/L)	This work’s sensor (mmol/L)	RSD (%)
#1	5.94	6.20	0.81
#2	4.77	5.00	1.00
#3	5.83	5.94	1.01
#4	4.33	4.61	1.30

### Special characteristics and performance of the prepared electrode

The electrochemical performance of the Ag@MoS_2_/GOA/W-AuE-based glucose sensor was compared with several previously reported non-enzymatic glucose sensors in terms of key performance metrics, including material composition, sensitivity, detection limit, linear range, stability, reproducibility, and potential applications. As shown in Table 2, the proposed sensor demonstrates exceptional performance across all these parameters.

The Ag@MoS_2_/GOA/W-AuE sensor developed in this study exhibits excellent stability, retaining 86% of its initial response after 30 days, along with high reproducibility (2.20% RSD). Additionally, its compact design enhances its suitability for implantable biomedical applications. These characteristics make it a strong candidate for long-term, non-enzymatic glucose detection in medical and implantable settings.

Compared to other non-enzymatic glucose sensors reported in the literature, the Ag@MoS_2_/GOA sensor demonstrates superior performance in terms of sensitivity, detection limit, and linear range. For instance, sensors based on CuO or NiO nanostructures often suffer from limited stability and selectivity, whereas the incorporation of AgNPs and MoS_2_ in our design significantly enhances both stability and selectivity. The unique three-dimensional structure of the graphene oxide aerogel (GOA) further contributes to the high surface area and efficient mass transfer, which are critical for achieving high sensitivity and low detection limits. These advantages make the Ag@MoS_2_/GOA sensor a promising candidate for practical applications in continuous glucose monitoring.

The mechanism of glucose detection in non-enzymatic sensors relies on the direct electrochemical oxidation of glucose on the surface of the electrode. In the case of the Ag@MoS_2_/GOA nanocomposite, the presence of silver nanoparticles (AgNPs) plays a crucial role in facilitating electron transfer due to their high electrical conductivity and catalytic activity. The MoS_2_ nanosheets provide a large surface area and active sites for glucose adsorption, while the graphene oxide aerogel (GOA) enhances the overall conductivity and stability of the electrode. The synergistic effect of these components results in a highly efficient glucose oxidation process, which is further amplified in alkaline conditions due to the presence of hydroxide ions (OH^−^) that promote the oxidation reaction.

## Conclusions

One-step hydrothermal method was successfully utilized to fabricate a highly sensitive non-enzymatic biosensor for glucose detection based on the Ag@MoS_2_/GOA nanocomposite. The morphological structure of Ag@MoS_2_/GOA provides a significant number of active sites for glucose electrooxidation. The nanocomposite was deposited by electrochemical polymerization onto a 1 cm layer at the beginning of the W/Au electrode wire. This method ensured strong adhesion of the catalytic layer to the electrode surface, preventing delamination during electrochemical testing, and uniform distribution of AgNPs and MoS_2__2_ nanosheets within the GOA matrix, maximizing active site exposure for glucose oxidation. The deposited layer maintained excellent structural stability in both alkaline (0.1 M NaOH) and physiological (PBS) environments, with no observable detachment or degradation during operation. The AgNPs@MoS_2_/GOA/W-AuE biosensor responds well to glucose concentrations ranging from 1.0 to 18.0 mM, with sensitivity and limit of detection (LOD) values of 24.70 μA mM^−1^ cm^−2^ and 0.52 µM, respectively. The AgNPs@MoS_2_/GOA-based glucose sensor exhibits exceptional selectivity against interfering species, acceptable repeatability, a wide linear range, and stability, indicating its potential as a non-enzymatic electrochemical glucose sensor. Silver nanoparticles are oxidized in the presence of the supporting electrolyte NaOH (0.1 M), promoting glucose redox reactions. The biosensor operates effectively in both alkaline and neutral (PBS) media; however, its performance is superior in an alkaline environment due to the enhanced electrocatalytic oxidation of glucose. To enable autonomous operation in compact configurations, an integrated Ag/AgCl reference electrode (IRE) was incorporated into the device architecture. This integration ensures reliable potential referencing without the need for external bulky electrodes, enhancing both portability and measurement stability.

### Outlook

The developed Ag@MoS_2_/GOA sensor shows promise for applications such as continuous glucose monitoring and fermentation process monitoring due to its high sensitivity, wide linear range, and stability in alkaline conditions. A key limitation for clinical use is its requirement for an alkaline environment, contrasting with the neutral pH of physiological systems. Future research should focus on optimizing material composition (e.g., AgNPs/MoS_2_/GOA ratios) to improve pH tolerance and exploring surface modifications or alternative nanomaterials for reliable operation in biological environments. Additionally, investigating its integration into wearable or implantable devices, considering long-term stability and biocompatibility, would be beneficial for practical applications.

## Data Availability

The datasets used and/or analysed during the current study available from the corresponding author on reasonable request.
